# Itaconate Attenuates Neuroinflammation and Exerts Dopamine Neuroprotection in Parkinson’s Disease through Inhibiting NLRP3 Inflammasome

**DOI:** 10.3390/brainsci12091255

**Published:** 2022-09-16

**Authors:** Guoqing Sun, Rui Zhang, Chengxiao Liu, Wenjun Meng, Qi Pang

**Affiliations:** 1 Department of Anesthesiology, Shandong Provincial Hospital, Shandong University, Jinan 250021, China; 2 Department of Neurosurgery, Shandong Provincial Hospital, Shandong University, Jinan 250021, China

**Keywords:** itaconate, Parkinson’s disease, NLRP3, inflammation, oxidative stress

## Abstract

Parkinson’s disease (PD) is a common age-associated neurodegenerative motor disorder, which is mainly caused by dopaminergic neuron loss in the substantia nigra. This study aimed to evaluate the function and the underlying molecular mechanism of itaconate in PD. PD models were established in vivo and in vitro using 1-methyl-4-phenyl-1,2,3,6-tetrahydropyridine (MPTP) and 1-methyl-4-phenylpyridinium (MPP^+^), respectively. Pole and rotarod tests were applied to evaluate the motor coordination of mice. The expression of tyrosine hydroxylase (TH) in MPTP-induced mice and the MPP^+^ revulsive PD cell model were detected using Western blotting and immunofluorescence. The inflammatory factors level was detected by quantitative real-time polymerase chain reaction. The content of superoxide dismutase (SOD), malondialdehyde (MDA), glutathione (GSH), and reactive oxygen species (ROS) in substantia nigra, striatum, and SH-SY5Y cells were analyzed. Moreover, the apoptosis of MPP^+^ revulsive SH-SY5Y cells was determined using terminal deoxynucleotidyl transferase-mediated dUTP-biotin nick-end labeling (TUNEL) staining and flow cytometry. The expression of apoptosis- and Nod-like receptor family protein 3 (NLRP3) inflammasome-associated proteins was measured using Western blotting and immunofluorescence. Itaconate attenuated motor deficits of MPTP-induced PD mice. Itaconate inhibited dopamine neuronal damage, inflammatory response, oxidative stress, and neuronal apoptosis in MPTP-caused PD mice and the MPP^+^ revulsive PD cell model. Additionally, itaconate notably repressed the activation of NLRP3 inflammasome. This research demonstrated that itaconate could attenuate neuroinflammation and exert dopamine neuroprotection in PD through inhibiting NLRP3 inflammasome.

## 1. Introduction

Parkinson’s disease (PD) is a common age-associated neurodegenerative motor disorder, which is mainly caused by dopaminergic neuron loss in the substantia nigra [1]. In China, approximately 4.9 million people are suffering from PD [2]. To date, no effective treatments can decelerate or stop the progression of PD. Hence, it is important to search for novel therapeutics for PD.

More and more studies have demonstrated that excessive oxidative stress, neuroinflammation, a-synuclein accumulation, and glial cell activation contribute to PD progression [3,4]. Itaconate is an unsaturated dicarboxylic acid, which has recently attracted extensive attention due to its role in inhibiting the inflammatory response [5]. Itaconate plays critical roles in macrophage metabolic remodeling and inflammation modulation by inhibiting succinate dehydrogenase [6]. Moreover, itaconate was reported to exert neuroprotective effects in the brain. Administration with itaconate increased the survival of mice experiencing traumatic brain injury [7]. Itaconate and its derivatives, for example, four-octyl itaconate [8] and dimethyl itaconate [9], benefit cortical neurons through the inhibition of oxidative stress and neuroinflammation. However, the role of itaconate in PD remains unclear.

The Nod-like receptor family protein 3 (NLRP3) inflammasome triggers inflammatory cascades that can be activated by a wide spectrum of stimuli, such as cytokines, lipopolysaccharides (LPSs), and virus infections [10,11]. In recent years, it has been found that the NLRP3 inflammasome exerts critical functions in the potential mechanisms of diverse inflammatory diseases, including Alzheimer’s disease, PD, and Huntington’s disease [12,13]. Que et al. [14] reported that Dl-3-n-butylphthalide can exert dopamine neuroprotection in PD through the inhibition of the NLRP3 inflammasome. A study by Yan et al. demonstrates that the inhibition of histone deacetylase 6 with tubastatin A exerts dopamine neuroprotection and reduces inflammation via inhibiting NLRP3 in PD [15]. In addition, itaconate confers tolerance to the late NLRP3 inflammasome as its accumulation upon prolonged inflammatory stimulation blocks the activation of caspase-1 and gasdermin D, which are essential for interleukin (IL)-1β and IL-18 secretion [11]. The inhibition of the NLRP3 inflammasome has been considered a potential mechanism of itaconate in treating multiple inflammatory diseases, for example, ischemia/reperfusion injury, pulmonary fibrosis, and peritonitis [16,17,18]. However, it is not clear whether itaconate plays a role in PD via modulating NLRP3 inflammasome.

In this research, we explored the function of itaconate in PD in vivo and in vitro and its underlying molecular mechanisms. The current study demonstrated that itaconate could attenuate neuroinflammation and exert dopamine neuroprotection in PD through inhibiting the NLRP3 inflammasome, proving that itaconate can be used as a promising therapeutic drug for treating PD in the future.

## 2. Materials and Methods

### 2.1. Animal and PD Model Establishment

Male C57BL/6 mice (8-weeks old, 22–25 g) were obtained from Pengyue Experimental Animal Breeding Co., LTD (Jinan, China) and were housed at a temperature of 22 ± 1 °C (12 h light-dark cycle) with ad libitum access to food and water for 7 days. Mice were randomly divided into five groups (5 mice per group): Control, 1-methyl-4-phenyl-1,2,3,6-tetrahydropyridine (MPTP), and MPTP + Itaconate (5, 10, and 20 mg/kg) groups. The MPTP + Itaconate (5, 10, and 20 mg/kg) groups of mice received intraperitoneal injections of itaconate daily, while other groups of mice received the same volume of saline daily for 10 consecutive days. MPTP (30 mg/kg/day) was intraperitoneally injected into the mice in MPTP, MPTP + Itaconate (5 mg/kg), MPTP + Itaconate (10 mg/kg), and MPTP + Itaconate (20 mg/kg) groups for 5 consecutive days after itaconate injection from day 11 to day 15. The mice were killed following the behavioral tests. MPTP were supplied by Sigma-Aldrich (St. Louis, MO, USA). Itaconate (purity 99%) was purchased from Yuanye (Shanghai, China). The use of animals is based on the ethical guidelines for the Care and Use of Laboratory Animals and approved by Shandong Provincial Hospital.

### 2.2. Behavioral Test

The mice were put on the top of a rough-surfaced pole (diameter 10 mm and height 52 cm) and a pole test was conducted as previously described [19]. Time taken by the mice to turn completely downward was recorded as the turn time. Additionally, the time required for the mice to climb down to the floor was recorded as the total time. Mice were placed on the rod with an accelerated speed from 5 rpm to 30 rpm within 60 s and kept at 30 rpm for 180 s [20]. Finally, the length of duration on the rod was recorded. The mice were pretrained on behavioral analysis once a day continuously for 10 days before MPTP treatment. The examiners were blind to the treatments.

### 2.3. Cell Culture and Treatment

Human neuroblastoma cell line SH-SY5Y was obtained from Procell (Wuhan, China) and was cultured at 37 °C in high-glucose Dulbecco’s Modified Eagle’s Medium (DMEM; Thermo Fisher, Waltham, MA, USA) containing 10% fetal bovine serum (FBS; AbsinBioscience, Shanghai, China) and 1% penicillin/streptomycin (Zeye, Shanghai, China) in a humidified atmosphere of 5% CO_2_. SH-SY5Y cells were pretreated with itaconate (25, 50, and 100 μM) for 24 h, and subsequently with 300 μΜ 1-methyl-4-phenylpyridinium (MPP^+^; Sigma-Aldrich, St. Louis, MO, USA) for 48 h.

### 2.4. 3-(4,5-Dimethylthiazol-2-yl)-2,5-Diphenyltetrazolium Bromide (MTT) Assay

MTT assay was applied for investigating SH-SY5Y cell viability. Cells were seeded into a 96-well plate and the cell viability was explored using MTT cell proliferation and a cytotoxicity assay kit (Beyotime, Shanghai, China) as described in a previous report [21].

### 2.5. Cell Apoptosis

SH-SY5Y cell apoptosis was investigated using an Annexin V-fluorescein isothiocyanate Apoptosis Detection kit (Beyotime, Shanghai, China). In brief, SH-SY5Y cells (3 × 10^5^) were resuspended and then double stained with AnnexinV-FITC and PI in a binding buffer. The apoptotic levels were assessed in a FACScan flow cytometer (BD Biosciences, San Jose, CA, USA).

### 2.6. Terminal Deoxynucleotidyl Transferase-Mediated dUTP-Biotin Nick-End Labeling (TUNEL) Staining

The Click-iT Plus TUNEL assay (ThermoFisher, Waltham, MA, USA) was applied to assess SH-SY5Y cell apoptosis following the manufacturer’s protocol. SH-SY5Y cells were quickly fixed with 4% paraformaldehyde (Sigma-Aldrich, St. Louis, MO, USA) and then permeabilized using 0.1% Triton X-100 (Leyan, Shanghai, China). TUNEL solution was subsequently added to SH-SY5Y cells in darkness for 30 min. Moreover, 4′-6-diamidino-2-phenyindole solution (Sigma, USA) was used to counterstain the cell nuclei for 15 min in darkness. Apoptotic cells were observed under a fluorescence microscope.

### 2.7. Intercellular Reactive Oxygen Species (ROS) Detection

ROS level was assessed using a ROS kit (Nanjing Jiancheng Bioengineering Institute, Nanjing, China) based on the manufacturer’s protocol. Briefly, SH-SY5Y cells were treated with DCFH-DA (5 μM) for 30 min in darkness. The fluorescence intensity of SH-SY5Y cells was determined with a FACScan flow cytometer.

### 2.8. Detection of Oxidative Stress-Related Factors

The content of superoxide dismutase (SOD), malondialdehyde (MDA), and glutathione (GSH) in the substantia nigra, brain striatum, and SH-SY5Y cells was detected using the corresponding commercial kits (Nanjing Jiancheng Bioengineering Institute) based on the manufacturer’s guidance.

### 2.9. Quantitative Real-Time Polymerase Chain Reaction (qRT-PCR)

Total RNA from the substantia nigra, striatum, and SH-SY5Y cells was collected by TRIzol total RNA extraction reagent (Nanjing Jiancheng Bioengineering Institute, China) and then reverse-transcribed into complementary DNA using a cDNA first chain synthesis kit (Nanjing Jiancheng Bioengineering Institute). qRT-PCR was carried using a UltraSYBR One Step RT-qPCR kit (Nanjing Jiancheng Bioengineering Institute, China). The specific primers were purchased from Tsingke (Beijing, China) and primer sequences are listed in Table 1.

### 2.10. Western Blotting Analysis

Total proteins from substantia nigra, striatum, and SH-SY5Y cells were extracted with a homogenization buffer using the RIPA lysis buffer (Beyotime, Shanghai, China). Protein (50 μg) was resolved on 10% sodium dodecyl sulfate–polyacrylamide gel electrophoresis (SDS-PAGE) and transferred on to a nitrocellulose membrane. Then, the membranes were incubated with primary antibodies overnight at 4 °C. After being washed with Tris-HCl-buffered saline with 0.1% (*v*/*v*) Tween 20 (TBST), the immunoreative bands were detected with horseradish peroxidase (HRP)-conjugated secondary antibodies for 1 h at room temperature and visualized using an enhanced chemiluminescent (ECL) kit (Beyotime, Shanghai, China). The primary antibodies were listed as follows: rabbit anti-tyrosine hydroxylase (TH; 1:5000, ab137869, Abcam (Cambridge, UK)), rabbit anti-Iba1 (1:1000, ab178846, Abcam), rabbit anti-cleaved caspase 3 (1:5000, ab214430, Abcam), rabbit anti-Bcl-2 (1:2000, ab182858, Abcam), rabbit anti-Bax (1:1000, ab32503, Abcam), rabbit anti-NLRP3 (1:1000, ab263899, Abcam), goat anti-apoptosis-associated speck-like protein (ASC; 1:1000, ab175449, Abcam), rabbit anti-pro-caspase 1 (1:1000, ab179515, Abcam), rabbit anti-caspase 1 (1:1000, ab207802 and ab138483, Abcam), rabbit anti-IL-1β (1:1000, ab283818, Abcam), rabbit anti-Nurr1 (1:1000, #10975-2-AP, Proteintech (Rosemont, IL, USA)), and mouse-anti-GAPDH (1:5000, #CL594-60004, Proteintech). The quantification of Western blot images was performed using Image J software (National Institutes of Health, Bethesda, MD, USA).

### 2.11. Immunofluorescence

The brain tissues were fixed in 4% paraformaldehyde for 48 h, embedded in paraffin, and then cut into 5 μm-thick sections. Meanwhile, the cells were fixed with paraformaldehyde and permeabilized using Triton X-100. Substantia nigra and striatum sections and cells were incubated with primary rabbit anti-TH (1:100, ab137869, Abcam), rabbit anti-NLRP3 (1:30, #19771-1-AP, Proteintech), or rabbit anti-ASC (1:100, ab283684, Abcam) antibodies at 4 °C overnight. Afterwards, slides were hatched with a secondary antibody bound to the fluorescein isothiocyanate for 60 min in the dark at room temperature. Nuclei were labeled with 4′,6-diamidino-2-phenylindole for 3 min. Fluorescent images were captured with a Nikon Eclipse Ni fluorescence microscope (Nikon, Tokyo, Japan) and analyzed using the Image J software.

### 2.12. Statistics

All of the data are presented as the mean ± SEM. Statistical significance was analyzed with one-way ANOVA followed by Tukey’s multiple comparisons test in SPSS software (version 22.0; SPSS Inc., Chicago, IL, USA) and GraphPad Prism 8.0. (GraphPad Software, Inc., San Diego, CA, USA) Significance was identified at *p*-value < 0.05.

## 3. Results

### 3.1. Itaconate Attenuated Motor Deficits and Dopamine Neuronal Damage in MPTP-Induced PD Mice

**Figure 1:**
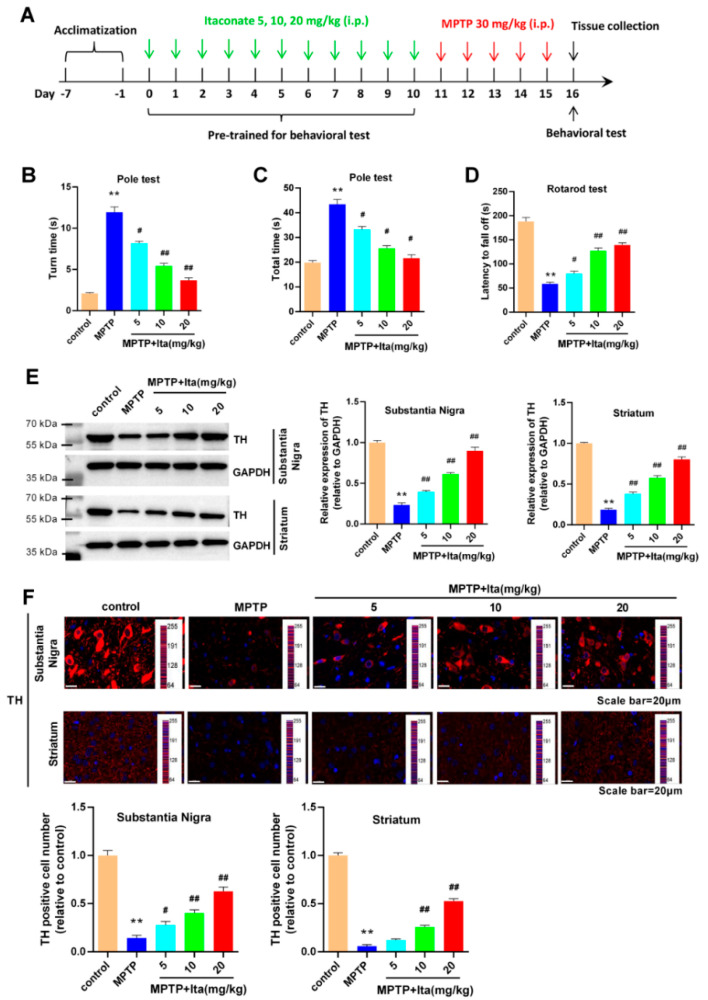
Itaconate attenuated motor deficits and dopamine neuronal damage in MPTP-caused PD mice model. (**A**) The experimental timeline of animal studies. Turn time (**B**) and total time (**C**) spent in the pole test. (**D**) Latency of falling off the rotarod. (**E**) The expression of TH in substantia nigra and striatum was measured using Western blotting. GAPDH serves as an internal control. (**F**) TH-positive cells in substantia nigra and striatum were assessed using immunofluorescence. The image acquisition parameters: objective (10×) and exposure settings (DAPI: 5 ms; TH: 200 ms). n = 5. ** *p* < 0.01 vs. control group; # *p* < 0.05, ## *p* < 0.01 vs. MPTP group. GAPDH, glyceraldehyde-3-phosphate dehydrogenase; Ita, Itaconate.

To evaluate the effects of itaconate on PD, an MPTP-induced PD animal model was established. The experimental timeline is shown in Figure 1A. The behavioral test showed that the mice in MPTP group needed more turns and total times (Figure 1B,C), and less time falling off the rotarod relative to the control group of mice (Figure 1D). Meanwhile, the effects of MPTP on mice behavior were partially reversed by the treatment of itaconate in a dose-dependent manner (Figure 1B–D). Subsequently, we detected TH expression in the substantia nigra and striatum by Western blotting and immunofluorescence. From the data shown in Figure 1E, it can be seen that MPTP significantly decreased the expression of TH in the substantia nigra and striatum of mice, while the low expression of TH was markedly reversed by itaconate treatment in a dose-dependent manner. Immunofluorescence results in Figure 1F confirm that MPTP-caused PD mice had a serious loss of TH-positive neurons and fibers in the substantia nigra and striatum, which was partially ameliorated after itaconate administration. All of the above data reveal that itaconate attenuated movement disorders and dopamine neuronal damage in MPTP revulsive PD mice.

### 3.2. Itaconate Attenuated Microglia Activation and Inflammatary Response in MPTP-Caused PD Mice

**Figure 2:**
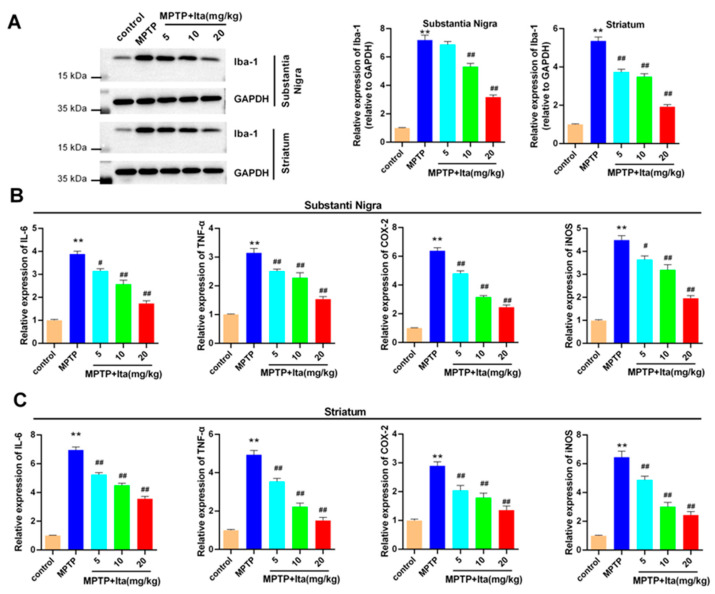
Itaconate attenuated microglia activation and inflammatory response in MPTP-caused PD mice model. (**A**) The expression of Iba-1 levels in substantia nigra and striatum were measured applying Western blotting. GAPDH serves as an internal control. The gene levels of IL-6, TNF-α, COX-2, and iNOS were detected with qRT-PCR in substantia nigra (**B**) and striatum tissues (**C**). n = 5. ** *p* < 0.01 vs. control group; # *p* < 0.05, ## *p* < 0.01 vs. MPTP group. GAPDH, glyceraldehyde-3-phosphate dehydrogenase; Ita, Itaconate.

The results in Figure 2A show that the Iba-1 expression was significantly higher in the MPTP group than in the control group. Meanwhile, the treatment of itaconate significantly inhibited the expression of Iba-1 induced by MPTP in the substantia nigra and striatum of MPTP mice (Figure 2A). Then, we further investigated the effects of itaconate on inflammatory genes. The gene levels of IL-6, TNF-α, COX-2, and iNOS were notably increased in the substantia nigra and striatum of MPTP-induced PD mice, which could be reversed by treatment with itaconate (Figure 2B,C). All results prove that itaconate attenuated microglia activation and inflammatory response in MPTP revulsive PD mice.

### 3.3. Itaconate Attenuated Oxidative Stress and Apoptosis in MPTP-Induced PD Mice

**Figure 3:**
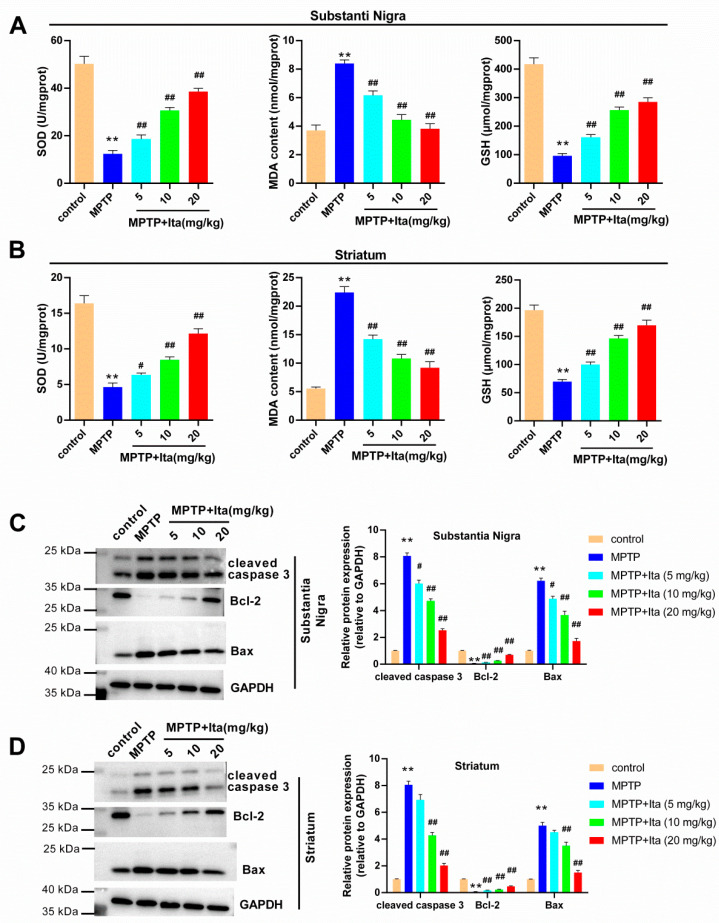
Itaconate attenuated oxidative stress and apoptosis in MPTP-induced PD mice. SOD, MDA, and GSH content were detected using their corresponding commercial kits in substantia nigra (**A**) and striatum (**B**). The protein expression of cleaved caspase 3, Bcl-2, and Bax in substantia nigra (**C**) and striatum (**D**) was analyzed using Western blotting. GAPDH serves as an internal control. n = 5. ** *p* < 0.01 vs. control group; # *p* < 0.05, ## *p* < 0.01 vs. MPTP group. GAPDH, glyceraldehyde-3-phosphate dehydrogenase; Bcl-2, B cell lymphoma-2; Bax, BCL-2 associated X; Ita, Itaconate.

As seen in Figure 3A,B, compared with the control group, the levels of SOD and GSH were decreased in the substantia nigra and striatum of the MPTP group of mice, and the MDA content was increased. This phenomenon was eradicated by treatment with itaconate. In addition, we explored the apoptosis-associated proteins using Western blotting analysis. The results verify that the protein levels of cleaved caspase 3 and Bax were elevated in the substantia nigra and striatum of MPTP-group mice, while Bcl-2 level was markedly reduced (Figure 3C,D). However, the protein levels of cleaved caspase 3 and Bax were lower in itaconate-group mice, while the Bcl-2 level was notably higher (Figure 3C,D).

### 3.4. Itaconate Repressed NLRP3 Inflammasome in MPTP-Caused PD Mice

**Figure 4:**
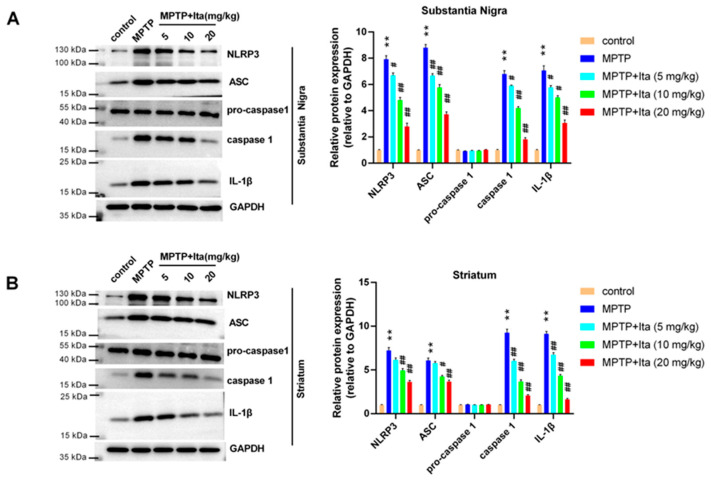
Itaconate inhibited NLRP3 inflammasome in MPTP-caused PD mice model. NLRP3, ASC, pro-caspase 1, caspase 1, and IL-1β in substantia nigra (**A**) and striatum (**B**) were measured using Western blotting. GAPDH serves as an internal control. n = 5. ** *p* < 0.01 vs. control group; # *p* < 0.05, ## *p* < 0.01 vs. MPTP group. GAPDH, glyceraldehyde-3-phosphate dehydrogenase; IL-1β, interleukin 1β; Ita, Itaconate.

Then, we explored the potential mechanisms of itaconate in MPTP-caused PD mice using Western blotting. As seen in Figure 4A,B, NLRP3, ASC, caspase 1, and IL-1β were highly expressed in the substantia nigra and striatum of the MPTP group of mice when compared with the mice in the control group. Itaconate treatment suppressed these expressions induced by MPTP (Figure 4A,B). These data show that itaconate inhibited the activation of NLRP3 inflammasome in the MPTP-caused PD mice model.

### 3.5. Itaconate Attenuated MPP^+^-Induced SH-SY5Y Cells Apoptosis

**Figure 5:**
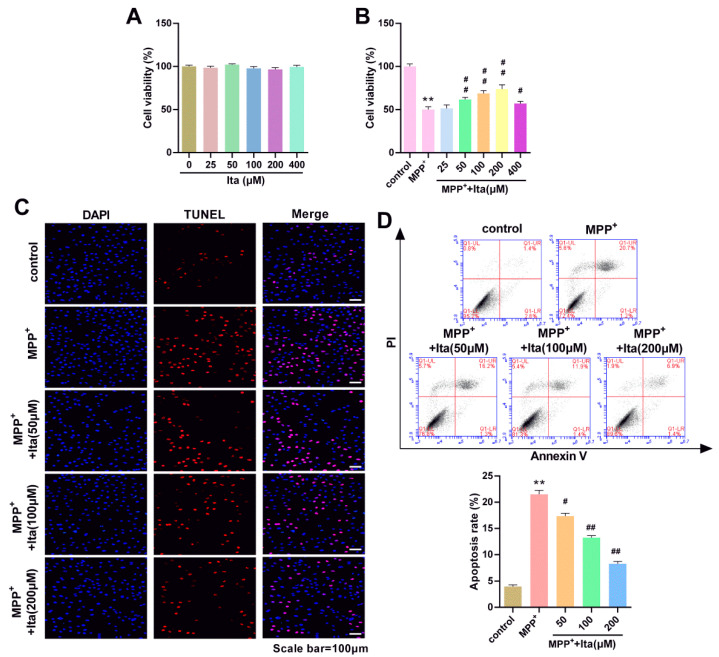
Itaconate attenuated the apoptosis in MPP^+^ revulsive PD cell model. (**A**) The effects of itaconate (from 0 to 400 μM) on SH-SY5Y cell viability were analyzed by MTT assay. (**B**) After treatment with MPP^+^, the function of itaconate on SH-SY5Y cell viability was detected by MTT assay. Following MPP^+^ treatment, the functions of itaconate on SH-SY5Y cell apoptosis were determined with TUNEL (**C**) and flow cytometry (**D**). n = 3. ** *p* < 0.01 vs. control group; # *p* < 0.05, ## *p* < 0.01 vs. MPP^+^ group. Ita, Itaconate.

Next, the effects of itaconate on MPP^+^-treated SH-SY5Y cells were explored. Figure 5A showed that itaconate (from 0 to 400 μM) did not affect SH-SY5Y cell viability. The viability of SH-SY5Y cells was inhibited after the treatment of MPP^+^ (Figure 5B). However, this phenomenon was significantly reversed by itaconate (50, 100, 200, and 400 μM) (Figure 5B). Itaconate at concentrations of 50, 100, and 200 μM was used in the following experiments. Subsequently, we thoroughly explored the function of itaconate on the apoptosis of MPP^+^ revulsive SH-SY5Y cells using TUNEL (Figure 5C) and flow cytometry (Figure 5D). Both TUNEL and flow cytometry results show that the apoptosis of SH-SY5Y cells was significantly increased in the MPP^+^ group (Figure 5C,D). When compared with the MPP^+^ group, the apoptosis of SH-SY5Y cells was dramatically reduced after being treated with itaconate. These data indicate that itaconate attenuated MPP^+^-induced SH-SY5Y cell apoptosis.

### 3.6. Itaconate Attenuated Dopamine Neuronal Damage in MPP^+^-Induced SH-SY5Y Cells

**Figure 6:**
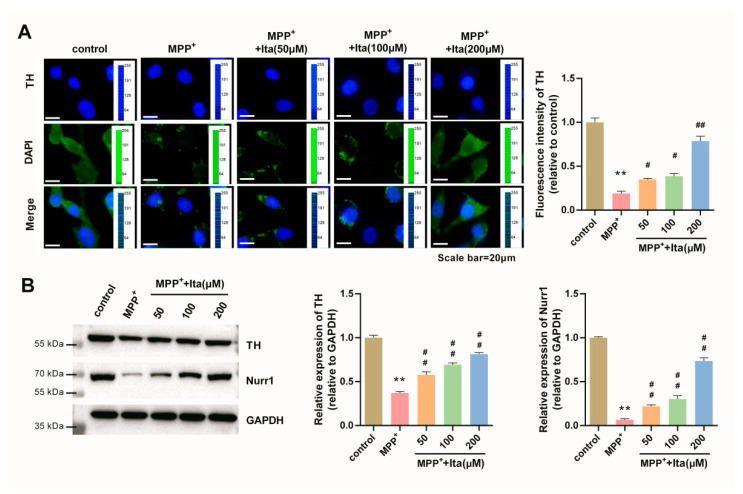
Itaconate attenuated dopamine neuronal damage in MPP^+^ revulsive PD cell model. (**A**) The expression of TH was analyzed by immunofluorescence (magnification, ×400). The image acquisition parameters: image resolution (−4908 × 3264), objective (10×), and exposure settings (DAPI: 5 ms, TH: 200 ms). (**B**) The expression of TH and Nurr1 in MPP^+^ revulsive PD cell model was measured by Western blotting. GAPDH serves as an internal control. n = 3. ** *p* < 0.01 vs. control group; # *p* < 0.05, ## *p* < 0.01 vs. MPP^+^ group. GAPDH, glyceraldehyde-3-phosphate dehydrogenase; Ita, Itaconate.

The immunofluorescence image showed that TH level was notably lower in MPP^+^-induced SH-SY5Y cells (Figure 6A). Meanwhile, itaconate treatment significantly reversed the reduced expression of TH caused by MPP^+^ (Figure 6A). Similarly, Western blotting results confirm that the expression of TH and Nurr1 was reduced in MPP^+^-treated SH-SY5Y cells, which was partly remitted by itaconate (Figure 6B). All data reveal that itaconate attenuated dopamine neuronal damage in MPP^+^ revulsive SH-SY5Y cells.

### 3.7. Itaconate Attenuated Oxidative Stress and Inflammatory Response in MPP^+^-Induced SH-SY5Y Cells

**Figure 7:**
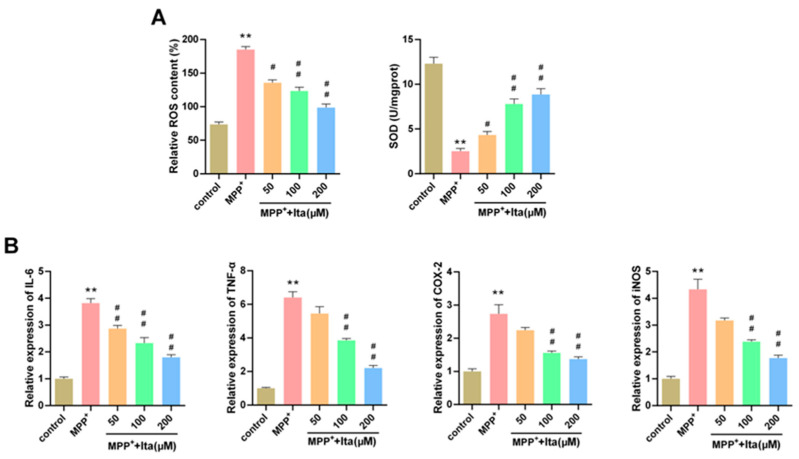
Itaconate attenuated oxidative stress and inflammatory response in MPP^+^ revulsive PD cell model. (**A**) ROS and SOD level in MPP^+^ revulsive PD cell model. The image acquisition parameters: image resolution (−4908 × 3264), objective (10×), and exposure settings (DAPI: 5 ms; TH: 200 ms). (**B**) IL-6, TNF-α, COX-2, and iNOS levels in MPP^+^ revulsive PD cell model were detected using qRT-PCR. n = 3. ** *p* < 0.01 vs. control group, # *p* < 0.05, ## *p* < 0.01 vs. MPP^+^ group. Ita, Itaconate.

As seen in Figure 7A, the ROS level was higher in the MPP^+^ group, while the SOD level was lower. This phenomenon was dramatically reversed by itaconate treatment (Figure 7A). The levels of IL-6, TNF-α, COX-2, and iNOS were notably increased in MPP^+^-induced SH-SY5Y cells, which could be reversed by treatment with itaconate (Figure 7B). Together, the data demonstrate that itaconate attenuated oxidative stress and inflammatory response in MPP^+^-induced SH-SY5Y cells.

### 3.8. Itaconate Inhibits NLRP3 Inflammasome in MPP^+^-Caused SH-SY5Y Cells

**Figure 8:**
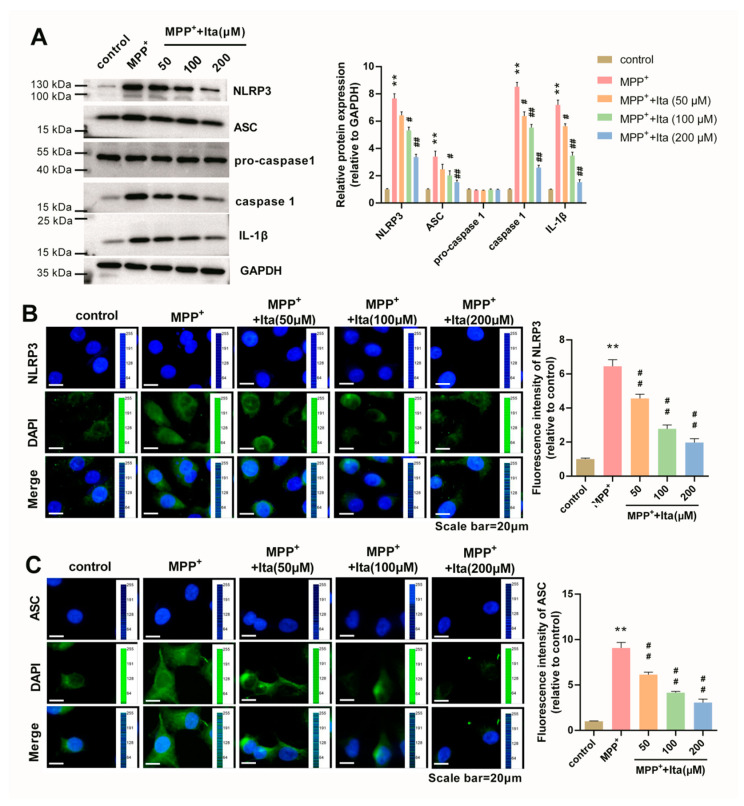
Itaconate inhibited the activation of NLRP3 inflammasome in MPP^+^ revulsive PD cell model. (**A**) The expression of NLRP3, ASC, pro-caspase 1, caspase 1, and IL-1β in MPP^+^ revulsive PD cell model was measured using Western blotting. GAPDH serves as an internal control. (**B**) The expression of NLRP3 in MPP^+^-induced SH-SY5Y cells was analyzed by immunofluorescence (magnification, ×400). (**C**) The expression of ASC in MPP^+^-induced SH-SY5Y cells was analyzed by immunofluorescence. The image acquisition parameters: image resolution (−4908 × 3264), objective (10×), and exposure settings (DAPI: 5 ms, NLRP3: 300 ms, ASC: 100 ms). n = 3. ** *p* < 0.01 vs. control group; # *p* < 0.05, ## *p* < 0.01 vs. MPP^+^ group. GAPDH, glyceraldehyde-3-phosphate dehydrogenase; IL-1β, interleukin 1β; Ita, Itaconate.

Figure 8A shows that NLRP3, ASC, caspase 1, and IL-1β were highly expressed in MPP^+^-treated SH-SY5Y cells. Itaconate treatment inhibited these expressions induced by MPP^+^ (Figure 8A). Additionally, the immunofluorescence results show that the expression of NLRP3 and ASC was dramatically elevated after MPP^+^ treatment, which was partly eradicated by itaconate (Figure 8B,C). All these data confirm that itaconate inhibited NLRP3 inflammasome in the MPP^+^-induced PD cell model.

## 4. Discussion

In this study, we used the MPTP-induced PD mice model and MPP^+^-induced cell model to investigate the neuroprotective effect of itaconate. Our results prove that itaconate attenuated dopamine neuronal injury and apoptosis, inflammation, and oxidative stress in both MPTP-caused PD mice and the MPP^+^ revulsive PD cell model.

The motor deficits of PD are reported to be induced by dopaminergic neuronal death [22], and dopamine has been proved to exert critical functions in PD [23]. Our data prove that MPTP-caused PD mice and the MPP^+^ revulsive PD cell model exhibited the severe loss of TH-positive neurons, and the treatment by itaconate could reverse this situation. Increasing numbers of studies have demonstrated that excessive oxidative stress, neuroinflammation, and neuronal death are acknowledged as crucial factors in the progression of PD [3,4]. The neuroinflammatory response is an undeniable phenomenon in PD pathology [24]. By detecting the level of inflammation-associated genes in MPTP mice and cell models, we demonstrated that itaconate attenuated the inflammatory response in PD. Due to the important functions of oxidative stress in dopaminergic neuronal death, oxidative stress becomes another undeniable factor in PD pathology [25]. Oxidative stress is reported to reflect an imbalance between the excessive production of ROS and the body’s ability to defend against toxic functions via antioxidant defense systems, including GSH and SOD [26]. Our study confirms that itaconate partly reversed the inhibitory effect of SOD and GSH levels, and the promoting effect of the ROS level induced by MPTP or MPP^+^ in PD model. Furthermore, we evaluated the effects of itaconate on neuronal apoptosis, and found that itaconate attenuated neuronal apoptosis in the PD model. All these results indicate that itaconate may be a promising drug for PD therapy.

NLRP3 inflammasome consists of NLRP3, ASC, and pro-caspase 1 [27,28]. The activation of NLRP3 inflammasome accelerates the maturation of caspase 1 and the production of pro-inflammatory cytokines [29]. In recent years, the NLRP3 inflammasome has been proven to exert critical effects in the potential mechanisms of many inflammatory diseases, including PD [12]. For instance, Echinacoside has been reported to protect dopaminergic neurons through suppressing the NLRP3/caspase-1/IL-1β pathway in PD [30]. The repression of NLRP3 inflammasome by glibenclamide can attenuate dopaminergic neurodegeneration and motor deficits in paraquat- and maneb-induced mouse PD models [31]. A study by Li et al. [32] revealed that Genkwanin inhibited MPP^+^-induced cytotoxicity through repressing the NLRP3 inflammasome in PD. Recently, itaconate has attracted extensive attention because of its anti-inflammatory functions [5]. Therefore, we further investigated whether itaconate protected against PD via regulating NLRP3 inflammasome. The results of Western blotting and immunofluorescence confirmed that itaconate inhibited NLRP3 inflammasome in MPTP-caused PD mice and the MPP^+^ revulsive PD cell model.

There are limitations in the present study. Firstly, this is a single-time study and further multiple-time studies will help us to understand the time-dependent effects of itaconate in treating PD. Secondly, the effects of itaconate on microglial cells and astrocytes need to be studied to fully reveal the complexity of itaconate.

## 5. Conclusions

Despite the fact that itaconate has been reported to benefit multiple inflammatory diseases through the inhibition of NLRP3 inflammasome, we demonstrate the potential of itaconate in treating PD and its relation to modulating the NLRP3 inflammasome in PD for the first time. Itaconate is effective in attenuating neuroinflammation and exerting dopamine neuroprotection in PD through inhibiting NLRP3 inflammasome. These findings indicate that itaconate may be a promising candidate for the prevention and treatment of PD.

## Figures and Tables

**Table 1:** The sequences for qPCR primers used in this study.

Gene	Forward	Reverse
Mouse IL-6	5′-TCTTGGGACTGATGCTGGTG-3′	5′-TTGCCATTGCACAACTCTTTTC-3′
Mouse TNF-α	5′-ACTGAACTTCGGGGTGATCG-3′	5′-CCACTTGGTGGTTTGTGAGT-3′
Mouse COX-2	5′-GCTCAGCCAGGCAGCAAATC-3′	5′-CACCATAGAATCCAGTCCGGG-3′
Mouse iNOS	5′-CTCTAGTGAAGCAAAGCCCAACA-3′	5′-CACATACTGTGGACGGGTCG-3′
Mouse β-actin	5′-CACTGTCGAGTCGCGTCCA-3′	5′-CATCCATGGCGAACTGGTGG-3′
Human IL-6	5′-ACTCCTTCTCCACAAGCGCC-3′	5′-TCTTCTCCTGGGGGTACTGG-3′
Human TNF-α	5′-CCCATGTTGTAGCAAACCCT-3′	5′-GAGGTACAGGCCCTCTGATG-3′
Human COX-2	5′-CGCTCAGCCATACAGCAAAT-3′	5′-GTCCGGGTACAATCGCACTT-3′
Human iNOS	5′-GGAGACGGGAAAGAAGTCTCC-3′	5′-ACCCCAGGCAAGATTTGGAC-3′
Human β-actin	5′-GATTCCTATGTGGGCGACGA-3′	5′-AGGTCTCAAACATGATCTGGGT-3′

## Data Availability

All data in this manuscript used to support the findings of this study may be released upon application to the correspondence author.

## Abbreviations

Glutathione (GSH), malondialdehyde (MDA), Nod-like receptor family protein 3 (NLRP3), Parkinson’s disease (PD), reactive oxygen species (ROS), and superoxide dismutase (SOD).
